# Development of Optimal Conditions for Synthesis of Molecularly Imprinted Polymers for Effective Terbium Sorption

**DOI:** 10.3390/polym17101398

**Published:** 2025-05-19

**Authors:** Laura Agibayeva, Yevgeniy Melnikov, Ayakoz Berdaly, Ruslan Kondaurov

**Affiliations:** 1 Biochemical Engineering Department, International Engineering and Technological University, Al-Farabi Ave. 93a, 050060 Almaty, Kazakhstan; laura.agibaeva@kaznu.edu.kz (L.A.); kondaurov@kazetu.kz (R.K.); 2 Faculty of Chemistry and Chemical Technology, Al-Farabi Kazakh National University, Al-Farabi Ave. 71, 050040 Almaty, Kazakhstan; ayayashka.b@mail.ru

**Keywords:** molecular imprinting, molecular recognition, imprinting factor, synthesis protocol, sorption of metals, lead nitrate, terbium nitrate

## Abstract

Molecularly imprinted polymers (MIPs) as well as non-imprinted polymers (NIPs) were synthesized for selective sorption of lead and terbium. The ratio of raw monomers for the terbium–MIPs’ synthesis was optimized based on the results of the synthesis of lead–MIP. It was found that the molar ratio of template/monomer/monomer/cross-linker = 1:5:5:8 was the most accurate for successful synthesis of the target MIP. As a result, the yields of the MIP and NIP on terbium were 59.3% and 61.2%, respectively. The structure of the imprinted samples was determined by FTIR spectroscopy. SEM analysis of the imprinted structures showed that the Tb–MIP contained a large number of pores compared to the NIP. The size of these pores ranged from 0.779 μm to 1.874 μm. The results of sorption experiments showed that the adsorption efficiency of Tb–MIP was seven times higher than that of NIP: the sorption degree was 70.80% for MIP and 9.95% for NIP. The imprinting factor was calculated and was equal to 7.06. The sorption process was described by the Radushkevich and pseudo-second-order kinetic models. It was shown that sorption by NIP occurred with a fast saturation of a lower Tb concentration, and the MIP’s sorption passed slower and more efficiently. The desorption degrees of Tb–MIP and NIP were 90.15% and 52.67%, respectively.

## 1. Introduction

Nowadays, the problem of developing really effective sorbents has become a key issue for industry. In hydrometallurgy, sorption methods are widely used to extract metals from solutions (e.g., gold, copper, heavy metals, rare earth elements). So, the extraction of target metals is presented by a wide range of sorbents, such as inorganic sorbents, chelating polymers, ion-exchange resins/membranes, polymer hydrogels, interpolymer systems, and carbon-based sorbents [1,2,3,4,5,6,7,8,9,10,11,12]. However, these technologies have a number of significant problems that limit their effectiveness and economic feasibility. Such issues can be named as follows: low selectivity of sorbents; sensitivity to solution composition; limited capacity and kinetics of sorption; regeneration and disposal problems; high cost of modern sorbents; technological difficulties [13,14,15,16,17,18,19]. Regarding this, the extraction of target metals from water media (such as environmental lakes or technological solutions) requires a new approach to the development of sorbents. The use of polymers with molecular imprints has wide potential for solving various tasks related to the recognition and isolation of target molecules from complex mixtures, as well as wastewater treatment. Molecular imprinting, as well as an ionic imprinting technique, was successfully used for recognition and sorption of different metals [20,21,22,23].

Polymers with molecular imprints (MIPs) are synthetic materials engineered with highly selective binding sites that mimic natural molecular recognition systems. These polymers contain tailor-made cavities, functionalized with specific chemical groups designed to adsorb target molecules with high specificity [24,25,26]. MIP is proposed to be used in different application fields: medicine [27,28], pharmacy [29], water purification [30], catalysis [31,32], etc. MIPs are used effectively to detect and recognize a wide spectrum of tiny chemical compounds, metal ions, and large-structured molecules such as viruses, proteins, and cells. For example, molecularly imprinted polymers were efficiently applied in the separation of various active components from plants, including flavonoids, organic acids, alkaloids, phenylpropanoids, anthraquinones, phenolics, terpenes, steroids, and diketones [33]. For biomedical purposes, MIPs are used for separation and recognition of biological small molecules and biomacromolecules [34], as well as microorganisms [35] and biologically active substrates—in particular, steroidal systems [36] and proteins [37]. Based on the recognition of biomolecules, the imprinting technique has been widely used in biomarker detection, i.e., biosensors [38,39]. In comparison with general biosensors, imprinted biosensors are characterized by fewer non-specific recognition sites. The idea of using the imprinting approach for creating sensors is described in a variety of works [40,41,42,43,44]. Detection in such sensors is based on different physical signals, e.g., colorimetric [45], electrochemical [46,47,48], and electrochemiluminescence [49]. Authors mention the high sensitivity and selectivity of such systems as the main advantages of imprinted polymers. This makes MIPs relevant and promising materials for use in various fields of science and technology.

As mentioned above, the imprinting technique was used for selective recognition and separation of different metals. Among them, several works were devoted to the sorption of rare earth metals [50,51,52,53,54,55,56], which could be found in industrial wastewaters. Filtration, chemical precipitation, neutralization, chelating ion exchange, and adsorption have all been used to remove such ions [57,58,59,60,61]. The latter method is often selected because of its efficiency, controllability, selectivity, and low cost. Imprinted polymers show more efficiency in comparison with other traditional ion exchange resins. In the case of terbium, there are not so many research studies devoted to selective recognition of this metal. For example, Jianping Li and co-authors developed a molecularly imprinted polymer sensor for the highly sensitive and selective determination of ultra-trace Tb^3+^ [62]. The Tb^3+^–ethylenediaminetetraacetic acid complex was used as the template molecule and was incorporated into mono-6-mercapto-β-cyclodextrin to form a Russian-Matryoshka-structured molecule. This structure was developed as a sensor for quantitative determination of metal, but not like a sorbent for its recovery. So, studies on the development of MIPs (along with NIPs) for selective extraction of lead and terbium were carried out in this work.

The ultimate goal of the molecular imprinting process is to generate MIPs with high specificity and affinity for the template. In theory, any species can be chosen as a target molecule, but in fact, for various reasons, not all species can be directly imprinted [63]. In some cases, due to the inability to use a certain template for detection, a template with a similar structure is applied instead. The molecular imprinting process involves four key components [25]: template (the target molecule that defines the cavity structure); functional monomer (interacts with the template to form a complementary binding site); cross-linking agent (provides structural rigidity to the polymer matrix); porogen/solvent (creates porosity, allowing template removal and subsequent analyte access). During synthesis, the functional monomer polymerizes around the template in the presence of a cross-linker, forming a rigid three-dimensional network. After polymerization, the template is removed, leaving behind cavities that precisely match the size, shape, and functional group arrangement of the target molecule. These “molecular memory” sites enable MIPs to selectively rebind the template or structurally similar compounds [24]. The sorption capacity and binding specificity of MIPs are highly dependent on the quantity, ratio, and type of reagents used in their synthesis. Therefore, selecting an optimal reagent system is crucial for achieving high-performance MIPs [64,65,66]. Therefore, the aim of this work is the development of optimal conditions for the synthesis of MIPs on lead and terbium and carrying out laboratory tests on the sorption effectiveness in relation to terbium.

## 2. Materials and Methods

### 2.1. Materials

#### 2.1.1. Templates

*Lead(II) nitrate* produced by “Sigma-Aldrich” (Burlington, MA, USA) with a composition of main product ≥ 99% was used without pre-treatment.

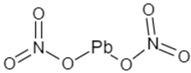



*Terbium(III) nitrate pentahydrate (Tb)* in a powder form with a composition of main product ≥ 99.9% produced by “ChemCraft” (Kaliningrad, Russia) was used without pre-treatment.

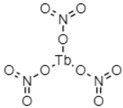



#### 2.1.2. Initiator

*Azobisisobutyronitrile* (AIBN) produced by “Sigma-Aldrich” (Burlington, MA, USA) in a powder form with a composition of main product ≥ 98% was used without pre-treatment.

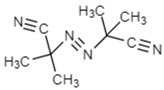



#### 2.1.3. Monomers

*Methacrylic acid (MAA)* produced by “Fluka Chemicals” (Taufkirchen, Germany) was used without pre-treatment with a purity ≥ 98%.

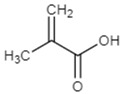



*2-Vinylpyridine (2-VP)* with a composition of main product ≥ 97% produced by “Sigma-Aldrich” (Burlington, MA, USA) was used without pre-treatment.

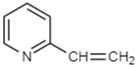



#### 2.1.4. Cross-Linker

*Divinylbenzene (DVB)* with a technical grade of 80% produced by “Sigma-Aldrich” (Burlington, MA, USA).

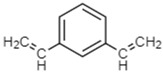



#### 2.1.5. Porogen

*Toluene* marked as “Pure for analysis” provided by “Skat” (Almaty, Kazakhstan) with a composition of main product ≥ 99.75% was used without pre-treatment.

#### 2.1.6. Stabilizer

*Hydroxyethyl cellulose (HEC)* in a powder form with an average molecular weight of 90,000 produced by “Sigma-Aldrich” (Burlington, MA, USA) was used without pre-treatment.

#### 2.1.7. Additional Materials

*Argon* was used for removing oxygen and making an inert surrounding.

*Dialysis membranes* with a pore size of 12–14,000 Daltons produced by “Medicell Membranes Ltd.” (UK) were used for purification of the imprinted polymers.

*Ethanol* produced by “TalgarSpirt” (Almaty, Kazakhstan) was used without pre-treatment with a composition of main product ≥ 96%.

*Nitric acid* produced by “Supelco” (Taufkirchen, Germany) with a composition of main product ≥ 98% was used without pre-treatment.

### 2.2. Synthesis of MIP and NIP

Synthesis of the molecularly imprinted polymer (MIP) and non-imprinted polymer (NIP) was carried out based on the following methodology [67].

*Synthesis of MIP on lead.* First, 1.656 g (5 mmol) of lead nitrate was dissolved in 10 mL of distilled water and in a certain amount of MAA and 2-VP in a three-necked round-bottom flask using a magnetic stirrer until the salt dissolved. The amount of monomers was determined according to the molar ratio of template/monomer-1/monomer-2/cross-linker (Table 1). Afterwards, 5 mL of toluene and 10 mL of 1% HEC solution were added, and the mixture was stirred for 15 min. Then, 5.697 mL of DVB and AIBN 1% by weight of the monomers were added and the mixture was stirred for 5 min. After that, the reaction mixture was blown up by argon for 5 min. The flask was placed in a water bath and kept for 6 h at 60 °C with constant stirring at 700–1000 rpm. The resulting mixture was centrifuged and divided into two parts. The upper oil layer was removed and the bottom water part was purified by dialysis using cellulose membranes within 4 days, the first day in ethanol, the second in distilled water, the third in a 0.05 M solution of nitric acid, and the last day in distilled water. Finally, the MIPs were freeze-dried.

*Synthesis of MIP on terbium.* First, 0.3 g (0.55 mmol) of terbium salt was dissolved in 10 mL of distilled water and in 0.233 mL of MAA and 0.298 mL of 2-VP in a three-necked round-bottom flask using a magnetic stirrer until the salt dissolves. Then, 5 mL of toluene and 10 mL of 1% HEC solution were added, and the mixture was stirred for 15 min. Afterwards, 0.627 mL of DVB and 0.0053 g of AIBN were added, and the mixture was stirred for 5 min. The mixture was blown up by argon for 5 min. Then, the flask was placed in a water bath and kept for 6 h at 60 °C with constant stirring at 700–1000 rpm. The resulting mixture was centrifuged. The water part was purified by dialysis during 4 days, the first day in ethanol, the second in distilled water, the third in a 0.05 M solution of nitric acid, and the last day in distilled water, and afterwards, freeze-dried.

*Synthesis of NIP.* The synthesis of NIP repeated the procedure for the synthesis of MIP but without using a template. In a three-necked round-bottom flask, 10 mL of distilled water and 0.233 mL of MAA and 0.298 mL of 2-VP were mixed using a magnetic stirrer. Then, 5 mL of toluene and 10 mL of 1% HEC solution were added, and the mixture was stirred for 15 min. Afterwards, 0.627 mL of DVB and 0.0053 g of AIBN were added, and the mixture was stirred for 5 min. The mixture was blown up by argon for 5 min. Then, the flask was placed in a water bath and kept for 6 h at 60 °C with constant stirring at 700–1000 rpm. The resulting mixture was centrifuged. The water part was purified by dialysis during 4 days, the first day in ethanol, the second in distilled water, the third in a 0.05 M solution of nitric acid, and the last day in distilled water. Afterwards, the NIPs were freeze-dried.

**Figure 1:**
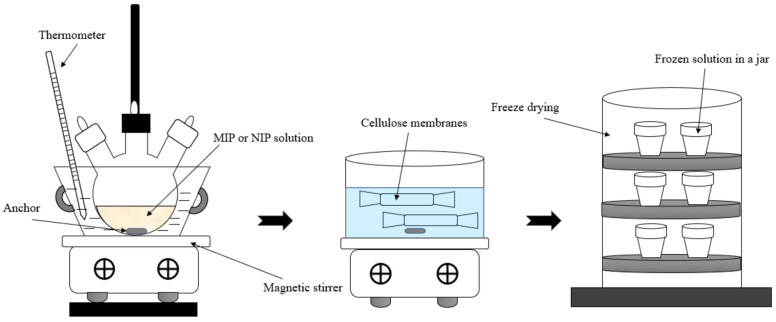
Synthesis of MIP and NIP.

In Figure 1, a methodological scheme of the synthesis of MIP and NIP is shown.

### 2.3. Physicochemical Methods of Analysis

The *freeze-drying method* was used in order to obtain dried MIP and NIP. For it, synthesized copolymer was frozen at around −20 °C and then dried using a Labconco 7934030 (Kansas City, MO, USA) freeze dryer.

*Fourier transform infrared (FT-IR) spectroscopy* was used to identify the chemical composition of the MIPs and NIPs. The FTIR spectra were received from a Nicolet iS10 FT-IR (Waltham, MA, USA) spectrometer in the region of 4000–450 cm^−1^. For this, samples of MIP and NIP were crushed into powder form.

A *scanning electron microscopy (SEM)* study was performed with a ZEISS Crossbeam 540 (Oberkochen, Germany) apparatus to identify the morphology of the object under study.

**Figure 2:**
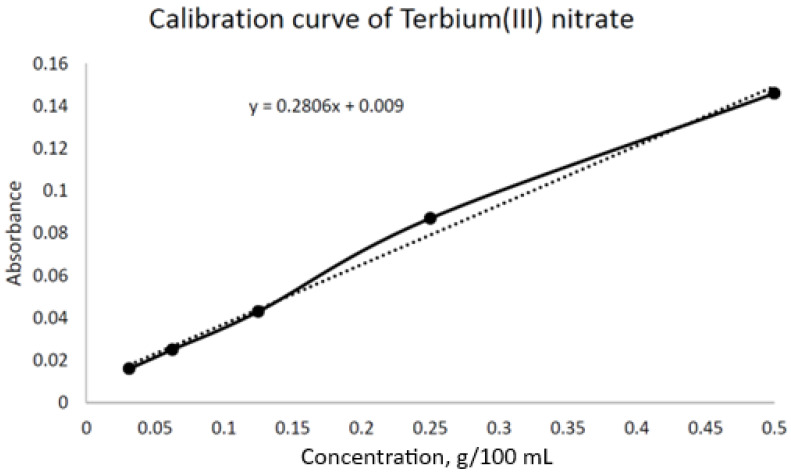
Calibration curve of terbium(III) nitrate.

The *adsorption method* was carried out in order to determine the adsorption capacity of the MIP and NIP. At first, the wavelength of the terbium solutions was determined using a concentration of 0.5 g/100 mL by a “Shimadzu UV-1900i” (Kyoto, Japan) UV–VIS spectrophotometer. Then, 5 types of solutions were prepared with different concentrations, like 0.03125, 0.0625, 0.125, 0.25, and 0.5 g/100 mL, and a calibration curve was built for terbium nitrate solutions with wavelengths of 281 nm (Figure 2). After that, 10 mL of the metal solutions were prepared with a concentration of 0.5 g/100 mL, and 0.01 g of MIP and NIP was placed into them in a glass filter. Then, using a “Shimadzu UV-1900i” (Kyoto, Japan) UV-VIS spectrophotometer, the absorbance of the solution was measured every 15 min throughout 3 h. After 24 h, a control measurement was carried out. The concentration of absorbed metal was found by subtracting the concentration of solution from the initial concentration.

The *desorption process* was carried out to define the release property of the polymer from its matrix. For this purpose, a weak solution of nitric acid was prepared with a concentration of 0.05 M (mol/L), and the samples obtained from the adsorption analysis were placed into beakers with 10 mL of this solution. As in the case of the adsorption analysis, the absorbance of the solution was measured every 15 min throughout 3 h with a wavelength for Tb of 281 nm. After 24 h, a control measurement was carried out. The concentration of desorbed metal was found by subtracting the concentration of solution from the absorbed concentration.

## 3. Results and Discussion

As highlighted in the literature, molecular imprinting functions like a miniature artificial “lock” designed to recognize a specific molecule, which acts as the corresponding “key” [68]. This technology enables researchers to create custom “locks” tailored to particular compounds. By treating the material, they form unique cavities that precisely match the target molecule in terms of the size, shape, and functional groups. A major advantage of these artificial receptors over natural ones is the flexibility in terms of the molecular design. Unlike natural receptors, which are typically limited to protein-based structures, artificial receptors can utilize a variety of frameworks, such as carbon chains or condensed aromatic rings, offering greater versatility.

The composition of MIP consists of some main reagents, such as monomers, initiators, cross-linkers, and porogens. The reason for choosing monomers for this research was based on work [69] where three types of sorbents were studied: using 2-VP (sorbent A), MAA (sorbent B) and both monomers taken in a molar ratio of 1:1 (sorbent C). During the synthesis of these sorbents in the presence of Boc-L-tryptophan (as a molecular template) and their subsequent use for the chromatographic separation of Boc-D,L-tryptophan racemate, it turned out that the highest values of the separation parameters were achieved on sorbent C, slightly lower—on sorbent A, and the lowest—on sorbent B. The authors believe that the presence of both monomers in the reaction mixture is necessary for the formation of a pre-polymerization complex that promotes the formation of selective adsorption centers. That is why MAA and 2-VP were chosen for the synthesis of MIP. Other components necessary were the following: initiator was AIBN, porogens were toluene and HEC, and cross-linker was DVB.

The use of AIBN as an initiator has several advantages; for example, it has a high reactivity and can initiate polymerization at low temperatures, which allows the production of polymers with a high degree of polymerization and molecular weight.

Toluene and HEC are used in the creation of MIP, because they have a certain structure that contributes to the formation of the desired porosity of the material during its synthesis, as well as the ease of removal from the resulting material [67].

In this work, MIP was obtained for the sorption of rare earth metals. To begin with, the synthesis scheme was worked out on lead, since rare earth metals are expensive.

### 3.1. Synthesis of the MIPs on Lead Nitrate

The formation of a stable complex between the monomer and the template molecules before polymerization is an essential requirement for the production of MIPs. That is why different molar ratios of raw materials were studied in order to obtain an effective MIP with stable bonding with the template. At first, the synthesis was carried out with proportions of 1:2:2:8 (template/monomer-1/monomer-2/cross-linker), but the polymer did not turn out. Then, synthesis was tried with 1:4:4:8, but the polymer structure turned out to be insufficiently porous. It was decided to increase the ratio to 1:5:5:8, and the result was positive. The results of these syntheses are summarized in Table 2. From these results, it can be concluded that the ratios of template and monomers of 1:2 and 1:4 are not enough to make MIP, i.e., the amount of monomers is not enough to make a pre-polymerization complex with a template.

**Figure 3:**
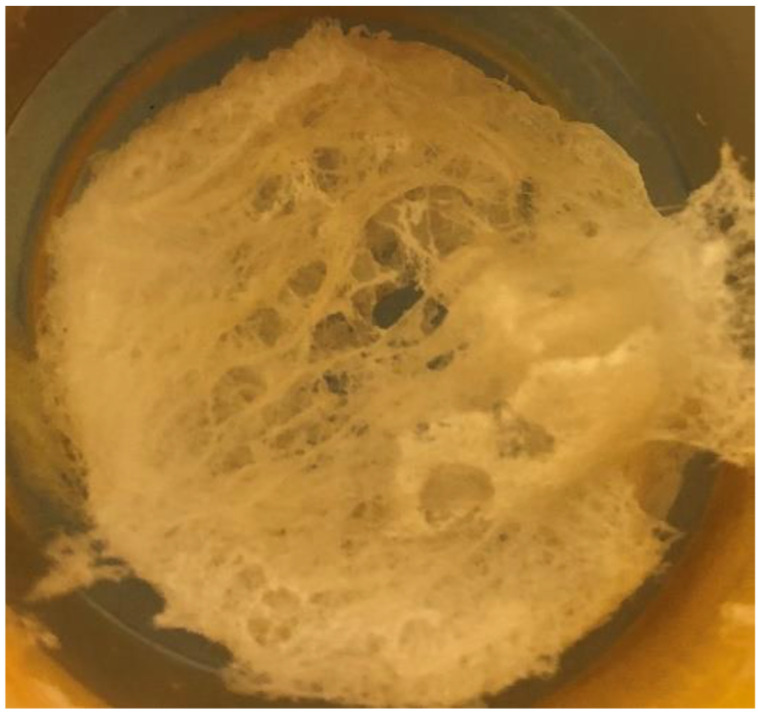
MIP on the basis of lead(II) nitrate with a 1:5:5:8 molar ratio of initial materials.

**Figure 4:**
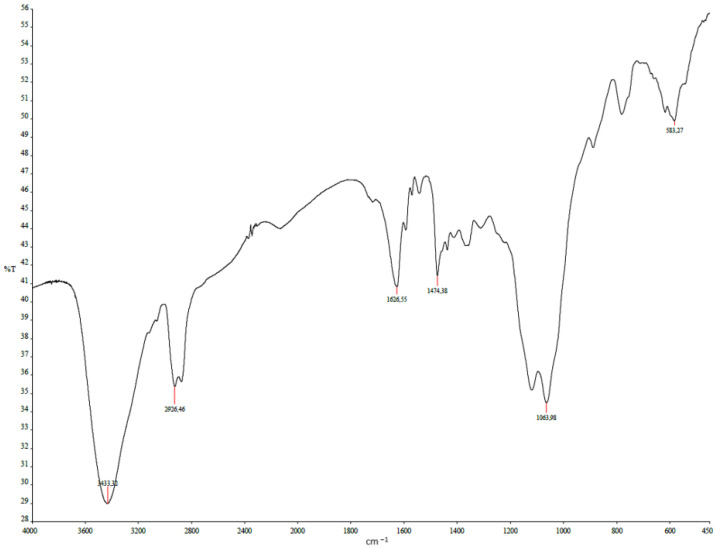
IR spectroscopy of the MIP of lead(II) nitrate.

Briefly, synthesis was carried out according to the following scheme. Firstly, the template lead(II) nitrate, monomers MAA and 2-VP, and cross-linker DVB were measured and weighed in a molar ratio of 1:5:5:8, respectively. Then, the template dissolved in distilled water and the monomers were mixed in a three-necked round-bottom flask using a magnetic stirrer until homogeneity. Afterwards, toluene and HEC were added as porogens and the mixture was stirred for 15 min. DVB and initiator AIBN were added. Then, the system was blown up by argon. The flask was placed in a water bath and kept for 6 h at 60 °C with constant stirring. The resulting mixture was centrifuged in order to separate the oil part from the water part. To remove the unreacted substances and template, the dialysis process was used. To accomplish this, the water part was poured into the cellulose membranes, making them into a shape similar to “sausages”. Then, dialysis was carried out for 4 days, the first day in ethanol, the second in water, the third in a weak solution of nitric acid, and the last day in water. The solvent for the dialysis was changed several times during a day for better purification of the copolymer. After this, the MIPs were freeze-dried. After freeze-drying, porous, white-colored cotton-wool-like structures were obtained, as shown in Figure 3. Along with this, an organoleptic analysis of the resulting MIP was carried out (Table 3). In order to study the structure of the obtained MIP, the samples were transferred to IR spectroscopy for analysis of the MIP composition. The results of the IR spectroscopy analysis are shown in Figure 4, and the description of the IR spectra is indicated in Table 4.

According to the obtained IR spectra (Figure 4) of the MIP on lead(II) nitrate, the following description can be made. The broad band with a peak at 3433.32 cm^−1^ could be defined as an -OH group with hydrogen bonding. The band at 1626.25 cm^−1^ is characteristic of carboxylate anion. Also, a narrow peak is observed at 1474.38 cm^−1^, which can be relative to an -CH_2_-CO- group. All these bands could characterize the functional group of methacrylic acid. The second monomer 2-VP could be defined in the IR spectrum by the intensive broad band at 1063.98 cm^−1^. The valence oscillation of the -CH_2_- group is usually in the form of several either weak or variable-intensity bands with a wavenumber of 2926.46 cm^−1^. The shifts of the peaks in the IR spectrum could be explained by the macromolecular structure of the obtained MIP and the possible steric effects of monomeric functional groups.

Thus, the synthesis at this molar ratio of 1:5:5:8 was successful, and further, the synthesis of Tb was carried out according to this developed procedure.

### 3.2. Synthesis of MIP on Terbium Nitrate

**Figure 5:**
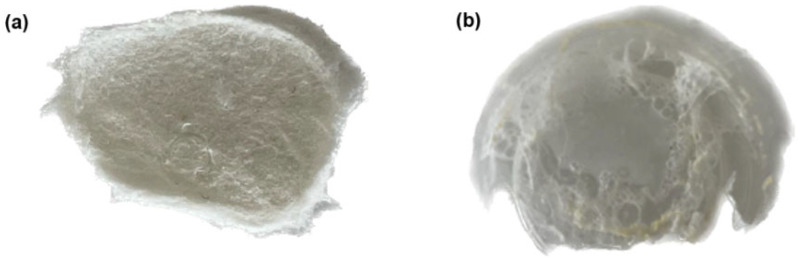
MIPs and NIPs after freeze-drying: (**a**) MIP of terbium; and (**b**) NIP of terbium.

After an optimal ratio was found for obtaining an imprinted porous structure, the MIP of terbium nitrate was synthetized by the methodology used for obtaining MIP on lead(II) nitrate. Synthesis of NIP was carried out in the same way as for MIP, but without a template. For this reason, the pores specific to the template were not formed. The MIP and NIP on terbium nitrate are shown in Figure 5.

According to the conducted organoleptic analysis, it can be noticed that the MIPs are spongier in structure and have a large number of cavities compared to the NIPs. Since a template is used in the synthesis of MIP, the polymers retain the shape and size of the template, and the NIP has cavities of different shapes, so the structure is denser and more heterogeneous, unlike the MIP, which has a homogeneous structure. They do not differ in color, as all the polymers are white.

#### 3.2.1. Yield of the Products

The yield of the reaction product is the ratio of the mass of the resulting product to the mass that should theoretically be obtained. The theoretical mass of the copolymers was taken as the total mass of the monomers used (Table 5).

The yield of the products was calculated by the following formula:

$$\omega =\frac{m_{pract.}}{m_{theoret.}}\times 100\%$$

Yield of the MIP of terbium:

$$\omega =\frac{0.312}{0.526}\times 100\%=59.3\%$$

Yield of the NIP of terbium:

$$\omega =\frac{0.325}{0.526}\times 100\%=61.2\%$$

As can be seen from the calculations, the yield of the products ranged from 59 to 62 percent, which is an average value. A possible reason why the yield was not more than 80 percent is because there is a possibility that active polymerization did not take place everywhere. The ratio of the monomers was taken based on the literature data and previously performed experiments on metals. Such an output is due to the fact that not all the monomers were able to enter the pre-polymerization complex. Accordingly, less product was obtained.

The difference between the MIP and NIP yields was around 3%, and the mass of the NIP final product was slightly larger than the MIP. This can be explained by the fact that not all the monomers were able to bind and form a pre-polymerization complex due to the steric effect, and the formed pre-polymerization complexes could not turn into MIP.

Also, another reason could be that the synthesis of the MIP may occur under conditions that are not always ideal for obtaining a high yield of the product. Various factors, such as the temperature and reaction time, can affect the yield of the product and lead to a low yield.

Since MIPs contain molecular imprints, they have a complex three-dimensional structure. During the synthesis process, MIP particles can bind to each other or to other particles of the material, which can lead to a decrease in the particle size and, as a consequence, to a decrease in the polymer yield.

Thus, the yield of the MIPs may be less than the theoretical yield due to various factors listed above.

**Figure 6:**
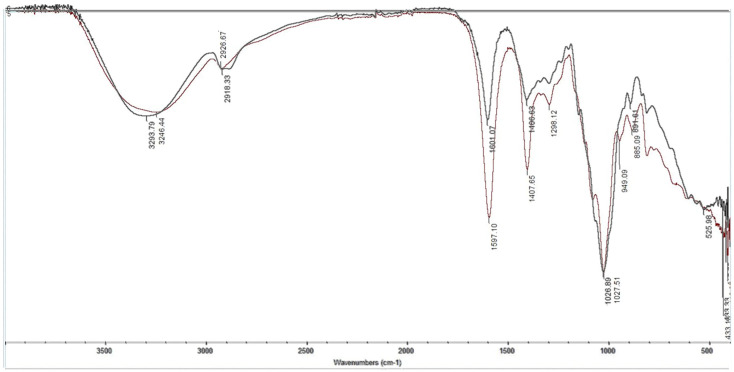
IR spectroscopy of the terbium(III) nitrate’s MIP (red line) and NIP (black line).

The obtained imprinted polymer structures were analyzed by IR spectroscopy, the results are shown in Figure 6 and the description is provided in Table 6.

According to the obtained IR spectra (Figure 6) of the MIP and NIP on terbium(III) nitrate, the following description can be offered. A broad intensive band is observed at 3246.44 and 3293.79 cm^−1^ for both samples. It could be defined as OH- groups; more exactly, intra- and intermolecular hydrogen bonding in the polymers. The valence oscillation of the -CH_2_- group is usually in the form of several either weak or variable-intensity bands with a wavenumber of 2956.93 cm^−1^ for Tb-MIP and 2918.33 cm^−1^ for NIP. For both samples, bands relative to the carboxylic group in MAA could be found on the IR spectrum: -COO^-^ at 1597.10 cm^−1^ for Tb-MIP and 1601.07 cm^−1^ for NIP; -CH_2_-CO- at 1407.65 cm^−1^ for Tb-MIP and 1406.63 cm^−1^ for NIP. All these band characterize the presence of MAA in the composition of the MIP and NIP. The presence of the second monomer 2-VP could be proved by the very intensive and narrow band at 1027.51 cm^−1^ for MIP and 1026.89 cm^−1^ for NIP, which is characteristic of a pyridine ring. Also, the band of 1,3-substitution in the benzene ring could be observed in the IR spectrum, which proves the presence of the cross-linker DVB in the imprinting structures.

**Figure 7:**
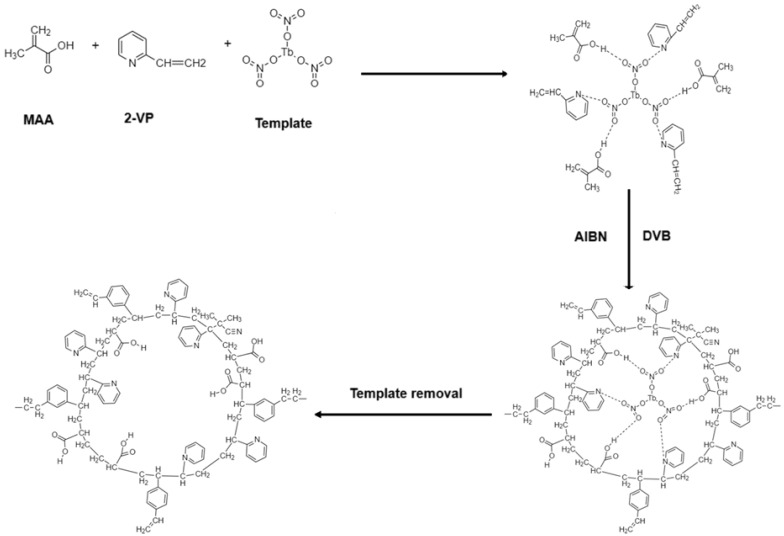
Schematic representation of the chemical reaction process for MIP production.

Based on the analysis of the IR spectra, as well as based on the literature data, it is possible to characterize how the reaction took place (Figure 7).

In the synthesis of the MIPs and NIPs, it is proposed that the method of non-covalent imprinting was used. In the non-covalent imprinting approach, a highly cross-linked polymer containing template molecules is generated during the polymerization of functional monomers with a large amount of cross-linking reagent in the presence of template molecules. At the first stage, to form a pre-polymerization complex, terbium nitrate is added to the mixture along with monomers to create molecular imprints. With non-covalent imprinting, the template binds with the monomers due to the hydrogen bond, the O⋯H bond is formed with MAA, and the N⋯H bond is formed with 2-VP. Then, an initiator AIBN is added to the mixture, which undergoes thermal decomposition with the formation of radical polymerization centers. Along with the initiator, a binding agent divinylbenzene is added, which just makes it possible to make cross-connections between the fingerprint and free functional groups of DVB.

As a result, the molecular imprints bind to each other and to the DVB, forming a network in which the molecular imprints remain permanent. In addition, DVB causes a narrowing of the pores in the structure, which increases the efficiency of extracting target molecules from the solution. After polymerization, the material is subjected to the extraction of a template that leaves empty spaces inside the polymer structure with the shape of a molecular imprint of terbium nitrate. The resulting polymer is separated from the remaining solution, and then, it is cyclically treated with some solutions to remove terbium and other undesirable impurities.

#### 3.2.2. Scanning Electron Microscopy

SEM provides an extensive view of the surface properties of the MIP material, including the size, shape, porosity and density. SEM can be used to analyze the quality of the MIP polymer, such as the damage, cracks in the structure, regularity and smoothness of the surface of the MIP material.

In addition, SEM analysis can help establish a connection between the features of the MIP surface and its properties, such as adsorption and desorption. This type of analysis can help develop more efficient methods for selectively extracting and separating target molecules from complex mixtures.

**Figure 8:**
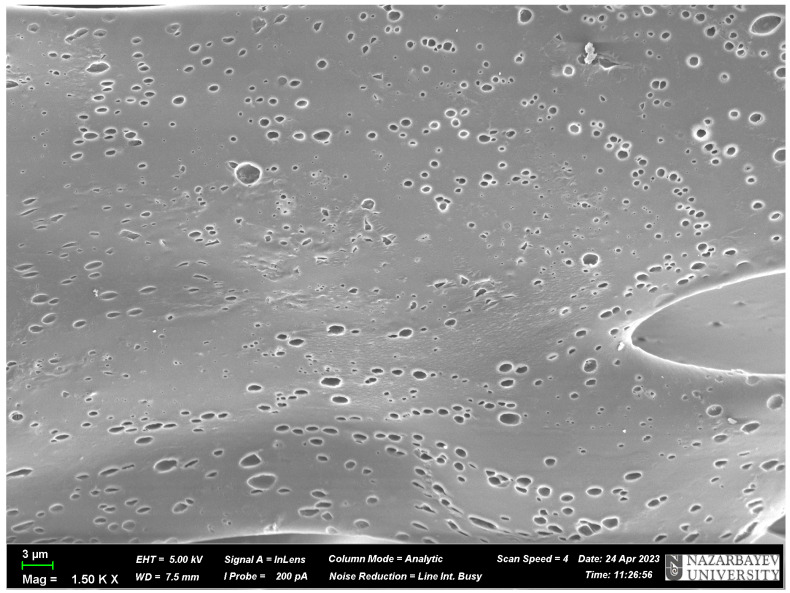
SEM picture of the MIP based on terbium(III) nitrate.

**Figure 9:**
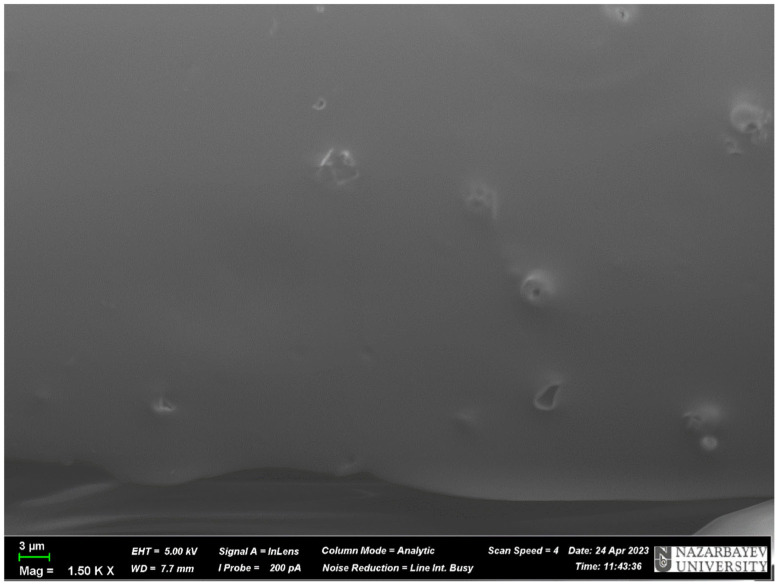
SEM picture of the NIP.

According to the obtained SEM pictures of the MIP (Figure 8) and NIP (Figure 9), it is visually noticeable that the MIP has more pores compared to the NIP. It indicates that the presence of the template (terbium salt) during the synthesis of the MIP leads to the occurrence of cavities (pores) in the structure of the polymer. The size of these pores ranges from 0.779 μm to 1.874 μm. This proves once again that the synthesis was successful.

#### 3.2.3. Sorption and Desorption Properties of the MIPs and NIPs

The sorption properties of the MIPs are based on their specific structure. These properties are explained by the processes of selective recognition of molecules in the presence of other molecules. It is caused by the presence of the molecular imprints that are created during the synthesis of the MIP polymer. The characteristic of the template determines the specificity, and hence, the ability of the MIP to adsorb specific molecules. Thus, the sorption and desorption properties of the MIPs ensure their use in various fields, such as chromatography, sensors, catalysts, membrane processes, etc.

In this regard, in this work, research was conducted on the adsorption and desorption of Tb from MIP and NIP. For this purpose, the wavelength of the solutions of terbium with a concentration of 0.5 g/100 mL was determined. The wavelength was 281 nm. After that, 10 mL of each metal solution was prepared, and the MIP and NIP were placed into them in a glass filter. Then, using a spectrophotometer, the absorbance of the solution was measured every 15 min throughout 3 h. After 24 h, a control measurement was carried out.

**Figure 10:**
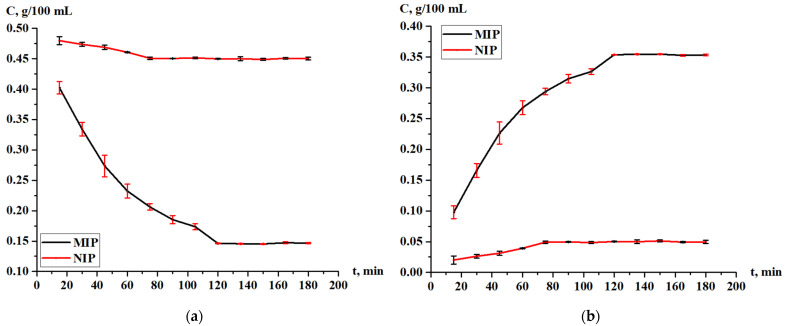
Change of the concentration of Tb during adsorption: in the solution studied (**a**); and in the MIP and NIP (**b**).

Along with the sorption of Tb on the MIP, the sorption on the NIP was also carried out on a comparative basis. The changing of the terbium concentration during sorption by the MIP and NIP is shown in Figure 10. From this graph, we can see that for the MIP, the sorption was active for 2 h and reached a concentration of 0.353 g/100 mL in a sorbent, and then, the value was constant. For the NIP, the sorption was active for more than 1 h, and after that, the value reached 0.050 g/100 mL in a sorbent and become constant. Based on the results in Figure 10, in order to evaluate the adsorption efficiency of the MIP and NIP, the sorption degree and sorption capacity were calculated.

The following equations were used for the calculations of the sorption parameters:

Sorption degree:

$$\eta =\frac{C_{0}-C_{equlibrium}}{C_{0}}\times 100\%$$

where C_0_ is an initial concentration of terbium nitrate in a solution, g/100 mL; and C_equilbrium_ is a concentration of terbium nitrate in a solution at a certain time interval, g/100 mL.

Sorption capacity:

$$Q=\frac{\left(C_{0}-C_{equlibrium}\right)\times V_{solution}}{m_{sorbent}}$$

where C_0_ is an initial concentration of terbium nitrate in a solution, g/100 mL; C_equilbrium_ is a concentration of terbium nitrate in a solution at a certain time interval, g/100 mL; V_solution_ is a volume of solution of terbium nitrate taken for sorption experiments, mL; and m_sorbent_ is a mass of MIP/NIP taken for sorption experiments, g.

**Figure 11:**
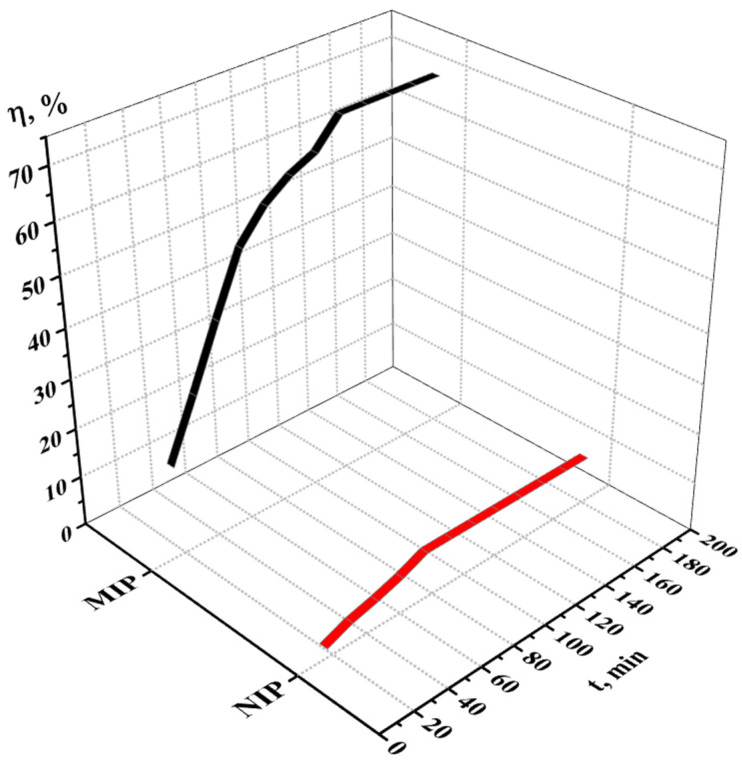
Sorption degree of the MIP and NIP in relation to Tb.

**Figure 12:**
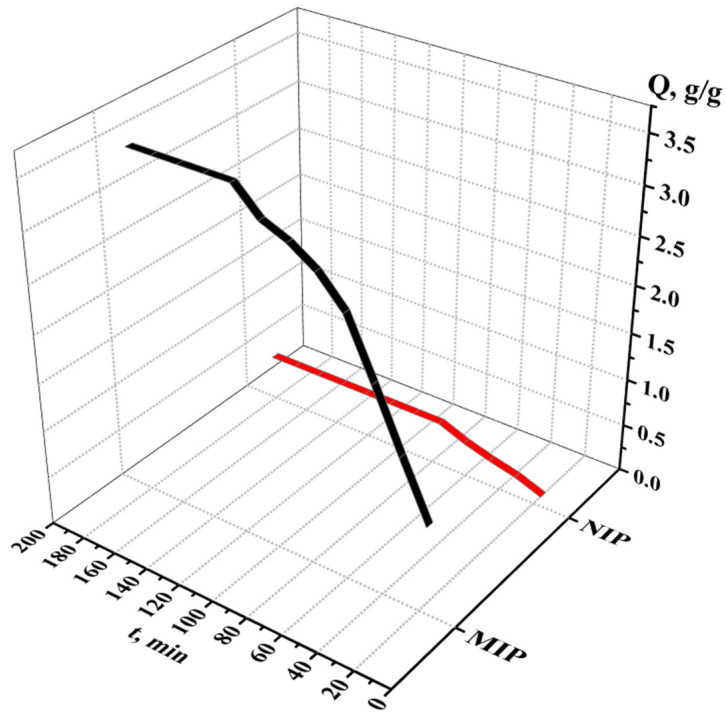
Sorption capacity of the MIP and NIP in relation to Tb.

The sorption degree of terbium on the MIP and NIP is shown on Figure 11, while the sorption capacity of the MIP and NIP is shown on Figure 12. From the data, we can conclude that the most active sorption is observed during 120 min (2 h); the sorption degree is 70.80% for MIP and 9.95% for NIP; and the sorption capacity is 3.54 g/g for MIP and 0.50 g/g for NIP. The Tb sorption character is different for MIP and NIP—in the case of NIP, the activity of sorption is observed at one hour, after what the values of the concentration and sorption properties become constant. Also, control measurements after 24 h were performed, and the values of the sorption parameters are presented in Table 7. Based on the results, it can be concluded that the adsorption efficiency of the MIP is seven times greater than that of the NIP.

Based on the obtained results, the imprinting factor (IF) was calculated [71]:

$$IF=\frac{Q_{MIP}}{Q_{NIP}}=\frac{Amount\;of\;target\;metal\;bound\;to\;the\;MIP}{Amount\;of\;target\;metal\;bound\;to\;the\;NIP}=\frac{0.353}{0.050}=7.06$$

For the calculations, the values of the Tb concentration in the MIP and NIP were taken. The IF is a key parameter used to evaluate the effectiveness of the synthetized MIP in selectively recognizing and binding the target molecule (template). It quantifies the enhanced binding affinity of the MIP compared to the NIP, which lacks the template-specific binding sites.

Interpretation of the IF values can be performed as follows [71]:

- IF = 1: No imprinting effect (MIP performs similarly to NIP).
- IF > 1: Successful imprinting (MIP has higher affinity for the template).
- IF < 1: Poor imprinting or non-specific binding issues.

It can be concluded that the synthesized MIP on Tb has a high affinity for the target molecule, and it correlates with the results of the sorption experiments and SEM images.

In order to evaluate the sorbents (MIP and NIP), the pseudo-second-order (PSO) kinetic model and Radushkevich kinetic model were used.

#### 3.2.4. Pseudo-Second-Order Kinetic Model

The non-linear PSO equation can be presented as follows:

$$q_{t}=\frac{k_{2}\times q_{e}^{2}\times t}{1+k_{2}\times q_{e}\times t}$$

where q_e_ is an equilibrium sorption capacity, mg/g; q_t_ is an amount adsorbed at time t, mg/g; and k_2_ is a PSO rate constant, g/(mg × min).

For the calculation of k_2_ and q_e_, the above-mentioned equation should be linearized:

$$\frac{t}{q_{t}}=\frac{1}{k_{2}\times q_{e}^{2}}+\frac{1}{q_{e}}\times t$$

**Figure 13:**
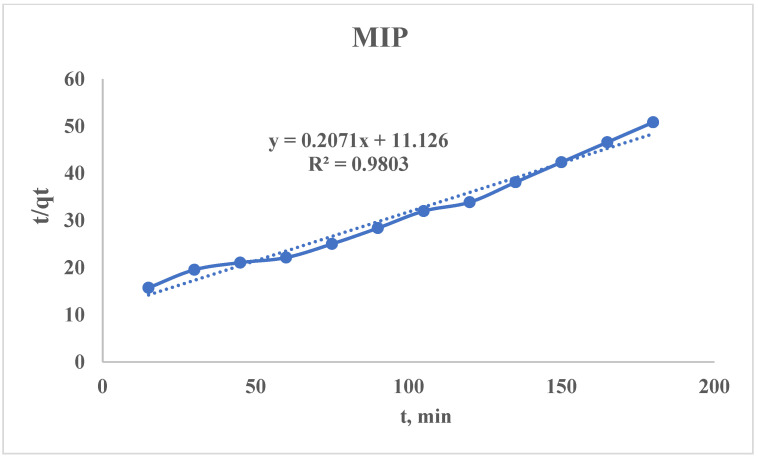
PSO kinetic model’s curve for the MIP.

**Figure 14:**
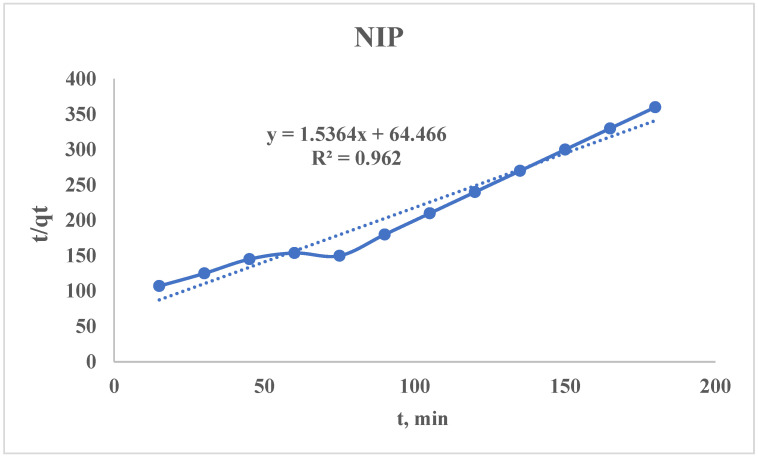
PSO kinetic model’s curve for the NIP.

Using the sorption data, the curve of t/q_t_ = f(t) is plotted (Figure 13 and Figure 14), from which the following parameters could be found:

$$Slope\left(S\right)=\frac{1}{q_{e}}$$

$$q_{e}=\frac{1}{S}$$

$$Intercept(I)=\frac{1}{k_{2}\times q_{e}^{2}}$$

$$k_{2}=\frac{1}{I\times q_{e}^{2}}$$

$$q_{e}(MIP)=\frac{1}{0.2071}=4.83\;mg/g$$

$$k_{2}(MIP)=\frac{1}{11.126\times 4.83^{2}}=0.0039\;\frac{g}{mg\times min}$$

$$q_{e}(NIP)=\frac{1}{1.5364}=0.65\;mg/g$$

$$k_{2}(NIP)=\frac{1}{64.466\times 0.65^{2}}=0.0366\;\frac{g}{mg\times min}$$

The obtained PSO kinetic rate constants for the MIP and NIP are realistic but seem counterintuitive at first glance. The higher values of k_2_ for the NIP means faster sorption (but this is not always better). For a proper comparison of the MIP and NIP sorption properties, it is necessary to calculate the initial sorption rate:

$$h=k_{2}\times q_{e}^{2}$$

$$h_{MIP}=0.0038\times 4.83^{2}=0.0887\;\frac{mg}{g\times min}$$

$$h_{MIP}=0.0366\times 0.65^{2}=0.0155\;\frac{mg}{g\times min}$$

Also, the time taken to reach 50% saturation (t_1/2_) should be calculated:

$$t_{1/2}=\frac{1}{k_{2}\times q_{e}}$$

$$t_{1/2(MIP)}=\frac{1}{0.0038\times 4.83}=54.4\;min$$

$$t_{1/2(NIP)}=\frac{1}{0.0366\times 0.65}=42.1\;min$$

The MIP has a higher effective rate than the NIP despite the MIP having a lower k_2_. The NIP reaches 50% saturation slightly faster (42.1 vs. 54.4 min), but its total capacity is 7.4 times lower (0.65 vs. 4.83 mg/g). The MIP sorbs slower per mass unit but removes far more target metal overall.

The NIP has a lower q_e_, which indicates that the adsorbent saturates quickly (low capacity); it may show a high k_2_ because equilibrium is reached faster, but the total uptake is poor. In other words, the NIP might bind adsorbates weakly but rapidly. It can be explained by binding to limited sites or weak surface interactions (high k_2_ + low q_e_), while in the case with MIP, slower but more extensive adsorption is observed (low k_2_ + high q_e_). Consequently, the NIP can be considered a sprinter (fast but tires quickly) while the MIP is a marathon runner (slower start but much higher total uptake).

For the evaluation of the sorbent from the PSO kinetic model point of view, the following should be taken in account:

(1) Joint comparison of h and q_e_ for the studied sorbents;
(2) High k_2_ ≠ “better”—it is only better if the q_e_ is also high.

#### 3.2.5. Radushkevich Kinetic Model

The non-linear type of Radushkevich equation can be presented as follows:

$$q_{t}=q_{e}\times (1-e^{-k_{R}\times t})$$

where q_e_ is an equilibrium sorption capacity (from the experimental data), mg/g; q_t_ is an amount adsorbed at time t, mg/g; k_2_—is a Radushkevich rate constant, min^−1^; and t is a time, min.

After linearization, the equation is:

$$\ln\left(1-\frac{q_{t}}{q_{e}}\right)=-k_{R}\times t$$

**Figure 15:**
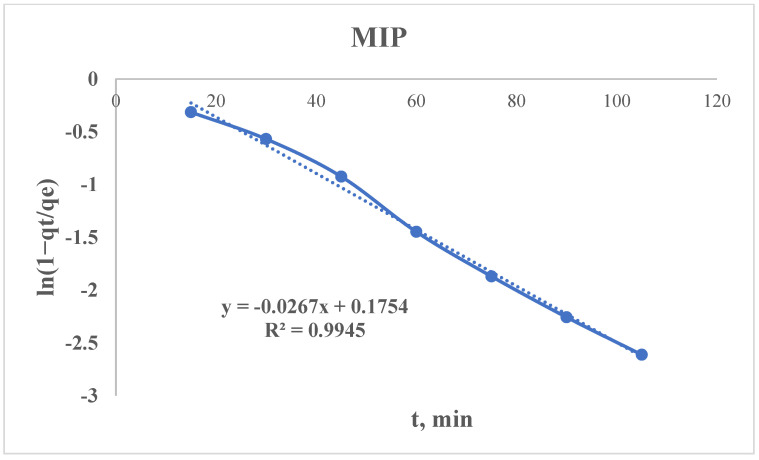
Radushkevich kinetic model’s curve for the MIP.

**Figure 16:**
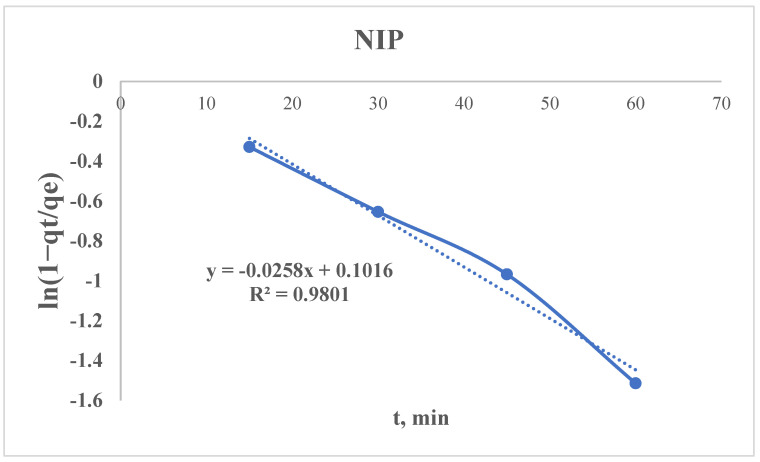
Radushkevich kinetic model’s curve for the NIP.

Using the sorption data, the curve of ln(1 − q_t_/q_e_) = f(t) is plotted (Figure 15 and Figure 16), from which the following parameter could be found:

$$k_{R}=-Slope\;(S)$$

$$k_{R}(MIP)=-\left(-0.0267\right)=0.0267\;{min}^{-1}$$

$$k_{R}(NIP)=-\left(-0.0258\right)=0.0258\;{min}^{-1}$$

A higher K_r_ means faster sorption kinetics—in our case, the MIP has a slightly higher K_r_ (0.0267 vs. 0.0258 min^−1^), which means that it reaches equilibrium marginally faster than the NIP.

As mentioned above, the PSO model suggested that the NIP has a higher k_2_ but much lower q_e_. The Radushkevich model shows similar K_r_ values, meaning the kinetics are comparable, but the MIP wins due to its higher capacity q_e_.

Kr reflects the diffusion rates in porous adsorbents—a higher K_r_ means faster diffusion into pores, while a lower K_r_ means slower diffusion (e.g., due to smaller pores or stronger adsorbate–adsorbent interactions).

But the Kr alone does not determine the “best” sorbent—the equilibrium capacity (q_e_) is a critical parameter.

Possible reasons why the MIP has a slightly higher K_r_:

1. Similar pore structures: If both sorbents have comparable porosity, their diffusion rates (K_r_) may differ only slightly.
2. Dominance of q_e_: The MIP may have more adsorption sites (higher q_e_), even if the kinetics are similar.

Also, for the evaluation of a material as a sorbent, desorption must be carried out along with adsorption. The desorption process shows the ability of the MIP to release the target molecules that were previously bound inside the polymer matrix. On the other hand, desorption means a process that allows the MIP to desorb the adsorbed molecules from its structure back into the reactive medium. At the same time, the specificity of the MIPs means that it is possible to extract ions for their further use and reuse them. To release bound molecules from the MIP, a solvent change desorption method was used. The work was performed with the same scheme as adsorption, but 0.05 M nitric acid was used as a solvent instead of water.

**Figure 17:**
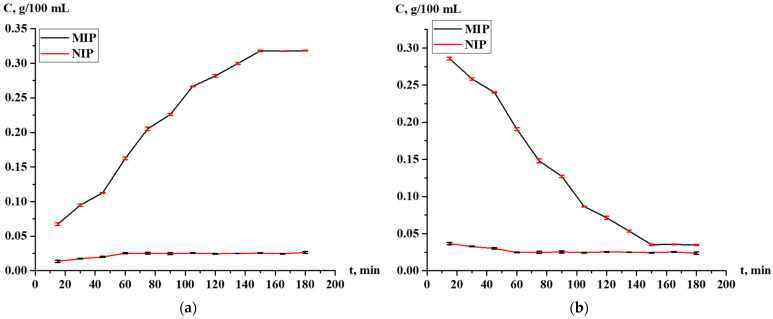
Concentration of Tb during desorption: in the solution studied (**a**); and in the MIP and NIP (**b**).

The change of the Tb concentration in the solution and in the MIP and NIP is shown in Figure 17. In the case with the MIP, the concertation of Tb increases in the solution (along with a decrease in the polymer) for 150 min (2.5 h), and after that time, desorption stops. At this time, the concentration of Tb in solution is 0.32 g/100 mL. The desorption of Tb from the NIP matrix occurs very slightly, constant values of the Tb concentration are observed after 60 min (1 h), and the concentration of Tb in the solution is 0.026 mg/100 mL. These values remain constant up to the control measurement after 24 h.

**Figure 18:**
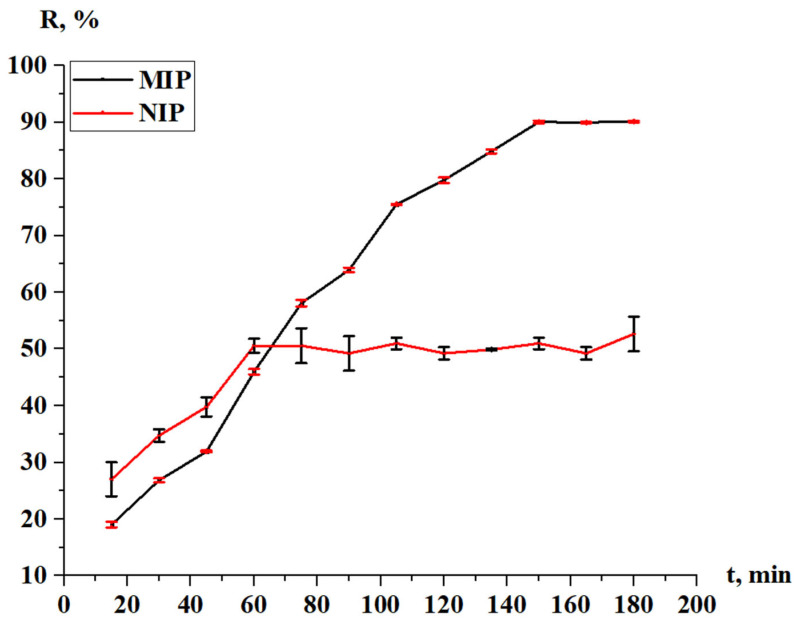
Desorption degree of Tb.

Figure 18 represents the desorption degree of Tb from the MIP and NIP. The desorption degree of Tb (MIP) increases sharply, reaching its final values 90.15% after 150 min, while this parameter for the NIP increases intensively for 1 h, after which the further increase is very slight—the final value of the parameter is 52.67%. In Table 8, the values of the desorption degree after 24 h are presented. Based on the results shown in Table 8, it can be concluded that the NIPs show low desorption properties compared to the MIPs.

Comparison of the sorption/desorption properties of the MIP and NIP shows that the MIPs have some advantages over the NIPs. The MIPs are able to selectively bind only the target molecules due to the molecular imprints that have the same shape and chemical properties of the analyte. The NIPs do not have such a level of specificity and can bind various molecules, which makes it difficult to use them in analysis and purification tasks. Due to their high selectivity, the MIPs can be more effective in binding the target molecules than the NIPs, since they can distinguish the target molecules from other components much more accurately. The MIPs have improved kinetic properties, such as faster and more uniform binding of the target molecules, which provides a faster purification process. Thus, the MIPs demonstrate improved sorption properties, which makes them more promising for application in various fields.

## 4. Conclusions

Based on the obtained data, the following conclusions can be drawn:

(1) The synthesis conditions for obtaining molecularly imprinted structures with lead and terbium salts as the templates are developed. The molar ratio of template/monomer/monomer/cross-linker = 1:5:5:8 is the most accurate for the successful synthesis of MIP along with NIP.
(2) MIPs contain large number of pores in comparison with NIPs, what is evidenced by the SEM analysis.
(3) A mechanism for obtaining MIP with a terbium template is suggested. The reaction is supposed to promote the hydrogen bonding of the salt part of rare earth metals with the carboxylic groups of methacrylic acid and the nitrogen groups of 2-vinylpyridine through the further polymerization of the monomers with the help of the cross-linker and initiator.
(4) The sorption effectiveness (in view of the studied sorption properties) of the developed MIP is more than seven times higher in comparison with the NIP. According to the PSO kinetic model, the NIP might bind adsorbates weakly but rapidly, while in the case with the MIP, slower but more extensive adsorption is observed.
(5) The desorption process of terbium from MIP and NIP allows extraction of 89.73% and 52.25% of the metal, respectively.

Based on the above-mentioned findings, it can be concluded that the developed MIPs can be considered promising new-generation sorbents for the selective sorption of target rare earth metals in industry.

## Figures and Tables

**Table 1:** The molar ratio of the monomers used for synthesis.

Ratio of Template/MAA/2-VP/DVB	Amount of Monomers, mL		Mass, mg
	MAA	2-VP	AIBN
1:2:2:8	0.848	1.085	19.1
1:4:4:8	1.696	2.17	38.2
1:5:5:8	2.12	2.712	47.7

**Table 2:** Comparative characteristics between different molar ratios.

Ratio of Template/MAA/2-VP/DVB	Description
1:2:2:8	Oily, non-porous
1:4:4:8	Dense, insufficiently porous
1:5:5:8	Spongy, porous

**Table 3:** Organoleptic analysis for lead nitrate MIP.

MIP of Lead Nitrate	
Color	White
Odor	Odorless
Consistency	Solid, cotton-like
Density	Low

**Table 4:** Characteristics of the IR spectrum frequencies for MIP of lead(II) nitrate.

Functional Group	Wavenumber, cm^−1^ (Theoretical Data) [70]	Wavenumber, cm^−1^
-OH	3550–3400	3433.32
-CH_2_-	2940–2915	2926.46
-COO^-^	1650–1550	1626.25
-CH_2_-CO-	1440–1400	1474.38
Pyridine ring	1100–1000	1063.98

**Table 5:** Theoretical mass of the products.

Sample	Mass of MAA, g	Mass of 2-VP, g	Total Mass, g
MIP of Tb	0.237	0.289	0.526
NIP of Tb	0.237	0.289	0.526

**Table 6:** Characteristics of the IR spectrum frequencies for terbium(III) nitrate.

Functional Group	Wavenumber, cm^−1^ (Theoretical Data) [70]	Wavenumber, cm^−1^ (MIP)	Wavenumber, cm^−1^ (NIP)
-OH	3400–3200	3246.44	3293.79
-CH_2_-	2940–2915	2926.67	2918.33
-COO^-^	1650–1550	1597.10	1601.07
-CH_2_-CO-	1440–1400	1407.65	1406.63
Pyridine ring	1100–1000	1027.51	1026.89
1,3-substitution	900–860	885.09	891.61

**Table 7:** Sorption parameters of the MIP and NIP in relation to Tb after 24 h.

Sorption Parameter	MIP	NIP
Sorption degree, %	70.80 ± 0.48	9.95 ± 0.07
Sorption capacity, g/g	3.54 + 0.02	0.50 ± 0.01

**Table 8:** Desorption degree of Tb from the MIP and NIP matrix after 24 h.

**Desorption degree, %**	**MIP**	**NIP**
	89.73 ± 0.16	52.25 ± 3.06

## Data Availability

The data presented in this study are available upon request from the corresponding author.

## Abbreviations

The following abbreviations are used in this manuscript:

MIP	Molecularly imprinted polymer
NIP	Non-imprinted polymer
SEM	Scanning electron microscopy
FTIR	Fourier transform infrared spectroscopy
IF	Imprinting factor
MAA	Methacrylic acid
2-VP	2-vinylpyridine
DVB	Divinylbenzene
EGDMA	Ethylene glycol dimethacrylate
HEC	Hydroxyethyl cellulose
AIBN	Azobisisobutyronitrile
